# Dosimetric and radiobiological comparison in different dose calculation grid sizes between Acuros XB and anisotropic analytical algorithm for prostate VMAT

**DOI:** 10.1371/journal.pone.0207232

**Published:** 2018-11-12

**Authors:** Kyeong-Hyeon Kim, Jin-Beom Chung, Tae Suk Suh, Sang-Won Kang, Seong-Hee Kang, Keun-Yong Eom, Changhoon Song, In-Ah Kim, Jae-Sung Kim

**Affiliations:** 1 Department of Biomedicine & Health Sciences, Research Institute of Biomedical Engineering, College of Medicine, the Catholic University of Korea, Seoul, Korea; 2 Department of Radiation Oncology, Seoul National University Bundang Hospital, Seongnam, Korea; North Shore Long Island Jewish Health System, UNITED STATES

## Abstract

To investigate feasible treatment planning parameters, we aimed to evaluate the dosimetric and radiobiological impact of the dose calculation algorithm and grid size in the volumetric modulated arc therapy (VMAT) plan for prostate cancer. Twenty patients were selected, and the treatment plans were initially generated with anisotropic analytical algorithm (AAA) and recalculated with Acuros XB (AXB) algorithm. Various dose grids were used for AXB (1, 2, and 3 mm) and AAA (1, 3, and 5 mm) plan. Dosimetric parameters such as homogeneity index (HI) and conformity index (CI), and radiobiological parameters such as tumor control probability (TCP) and normal tissue complication probability (NTCP) were calculated. Significant differences were observed in the planning target volume (PTV) coverage between both algorithms, and the V_95%_, HI, and CI of AAA were significantly affected by grid (p < 0.01). On 1 mm grid, the mean rectal dose difference between both algorithms was 2.87% of the prescription dose (p < 0.01), which was the highest among the critical organs. The TCP and NTCP of the AAA were higher than those of AXB (p < 0.01). Compared to AXB with 1 mm grid, the 2 mm grid showed comparable dose calculation accuracy with short calculation time. This study found that the PTV and rectum show significant differences according to dose calculation algorithm and grid. Considering the dose calculation performance for heterogeneous area, we recommend AXB with 2 mm grid for improving treatment efficiency of prostate VMAT.

## Introduction

Clinical use of volumetric-modulated arc therapy (VMAT) [1] has grown extremely since its debut in 2008. The application of VMAT for prostate cancer has been well-demonstrated for both plan quality and efficiency [2, 3]. A well-known advantage of VMAT is its ability to deliver faster treatment, which makes treatments patient-friendly and improves treatment accuracy because intrafractional motion is reduced. A previous study reported that VMAT produces comparable and higher conformal dose distribution and accurate dose delivery in comparison to intensity modulated radiotherapy (IMRT) [4–6].

In order to obtain effective treatment outcomes, minimal toxicity and complications should be achieved through reducing the radiation dose to organs at risks (OARs) near the prostate, such as the bladder, the femur, and the rectum. The endorectal balloon (ERB) for prostate radiotherapy can be implemented for sparing the rectal wall and reducing the intrafraction motion [7–10]. However, its use might introduce the heterogeneous region due to the generation of an air cavity in rectum, which will compromise the treatment efficacy, and therefore, accurate dose calculation is extremely important.

The Anisotropic Analytical Algorithm (AAA; Varian Medical Systems, Palo Alto, CA, USA) [11] is a convolution/superposition model for photon dose calculation. The AAA has improved accuracy of calculation compared to previous algorithms and is widely used at various treatment sites. The AAA calculates a dose accounting for lateral electron transport and, performs simplified density scaling of the kernels calculated in water. The AAA uses a kernel of water instead of a kernel with medium-specific characteristics, therefore it has limitation on accuracy of dose calculation. The Acuros XB (AXB; Varian Medical Systems, Palo Alto, CA, USA) [12], advanced dose calculation algorithm has been implemented for clinical use. The AXB uses the multiple-source model derived for the AAA, and performs dose calculation by explicitly solving the linear Boltzmann transport equation (LBTE). A mass density as well as material type was reflected to each given voxel in the AXB dose calculation. In other words, the AXB can reflect the radiation interaction according to each material property, and have been reported more accurate dose prediction in the heterogeneous region than the AAA in previous studies. Koo et al. [13] evaluated the dosimetric effect of AXB and AAA algorithms for prostate cancer and reported that dose calculation in the air cavity with AXB was more accurate than that of AAA. Kroon et al. [14] also investigated the performances of the dose calculation algorithm in the air cavity of non-small cell lung cancer (NSCLC). They have shown that the AXB with 2.5 mm calculated dose accurately, compared to the AAA. Kan et al. [15] analyzed dosimetric impacts of AXB and AAA on intensity modulated stereotactic radiotherapy (IMSRT) plans for nasopharyngeal carcinoma (NPC), and recommended the AXB as a standard reference for IMSRT boost for NPC. In most of studies, AXB showed generally low target coverage and OAR dose. In addition, previous studies have reported that dose distribution with AXB is more accurate and consistent with the Monte Carlo simulation in the heterogeneous region than that of AAA [16–18].

Since AAA uses the discretized kernel in lateral directions [19] and AXB also discretizes solution variables (space, angle, and energy) with material composition of voxels from the CT Hounsfield unit, both dose calculation algorithms are affected by the dose grid size. Although the error due to discretization may be smaller than the error obtained by the algorithm, the influence may be different depending on the algorithm and also be significant in some clinical cases. In general, the calculated dose distribution at fine grid is considered more accurately, however it is time consuming and may not feasible in the clinic. It is important to optimize the dose calculation algorithm and grid size at a reasonable calculation time without significantly compromising accuracy.

In other words, the calculated dose distributions of treatment planning system (TPS) are affected by the dose grid size, and the presence of a dosimetric influence according to the calculated grid size has been reported in IMRT and VMAT [20, 21]. Huang et al. [22] assessed the potential impact of grid size on dose calculation using AXB and AAA algorithms for stereotactic body radiotherapy for NSCLC, and showed that the dose difference between the two algorithms using a 2.5 mm grid was greater than that of a 1 mm grid on low density planning target volume (PTV). Previous studies have reported that the dose difference can be significant for radiobiological factors such as normal tissue complication probability (NTCP) and tumor control probability (TCP) [20, 23], which may affect radiotherapy quality and treatment success. Shiv P. Srivastava et al. [20] analyzed the radiobiological effects and dosimetric impacts of plans, according to calculation grid sizes, in head and neck cancers. They showed that a smaller calculation grid provides superior dosimetric outcomes with improved TCP and reduced NTCP.

In our previous study, we've confirm the AXB and AAA performance in a rectangular acryl phantom with an air cavity by EBT3 film dosimetry [13]. As a result, we confirmed that AXB had more similar results than AAA to film measurements in the air cavity and air-material interface in phantom. Unlike the phantom, patients have various anatomies, and the treatment plan for patient is more complicate than the phantom study. The influence of dose calculation grids and algorithms were not clearly identified in some clinical cases, and it is important for efficient patient care. No previous study has evaluated the impact on prostate VMAT plans by different dosimetric and radiological effects (TCP, NTCP) obtained by different algorithms under different grid sizes. In this study, we investigate the dosimetric and radiological impact on prostate VMAT plans with ERB from the dose calculation algorithms with different grid sizes.

## Materials and methods

### Patient selection and treatment planning

All experimental methods of this study were performed in accordance with the relevant guidelines and regulations. The Institutional Review Board of the Seoul National University Bundang Hospital approved the data collection and analysis (B-1802/451-106), and informed consent of the participants was waived by the IRB. All patient data used in the study were anonymized. Twenty patients who received prostate VMAT in our institution from April 2016 to April 2017 were randomly selected retrospectively. The average age of the patients was 64 (58−84) and the average weight was 73.1 kg (49.4−82.5 kg).

Prior to the planning computed tomography (CT) simulation, all patients were asked to drink 300 ml of water before the start of the one-hour of simulation, to ensure that their bladders were completely filled. An EBR was inserted into the rectum and inflated with approximately 70 cm^3^ with air. After 1 minute, the EBR was pulled toward the patient’s anal sphincter to the pre-marked position on the EBR catheter [24]. CT was performed using a Philips Big Bore CT scanner (Philips Medical Systems, Amsterdam, Netherlands).

The clinical target volume (CTV) on the planning CT was delineated from the prostate volume using magnetic resonance imaging. CTV included gross tumor and subclinical microscopic disease. The PTV was created from the CTV by expanding 5 mm posteriorly and 7 mm elsewhere. The rectum, bladder, left femoral head, and right femoral head were delineated as the OARs. Varian couch is modelled in our TPS and was inserted into each treatment plan (used for dose calculation).

The prostate VMAT plans were created using Eclipse TPS (version 11.0.34, Varian Medical System, Palo Alto, CA, USA) under the same dose prescription (78 Gy/39 fractions) and dose–volume criteria (Table 1). The aim of the planning optimization was to cover at least 95% of the PTV with 95% of the prescription dose. Two full arc techniques using a 10 MV beam from a Varian TrueBeam STX linear accelerator were used for optimal target coverage.

**Table 1:** Dose volume constraints for prostate volumetric modulated arc therapy plans.

Structure	Constraints
**Rectum**	V_30%_ < 7000 cGy
	V_50%_ < 5430 cGy
**Bladder**	V_30%_ < 7000 cGy
	V_50%_ < 5430 cGy
**Femoral heads**	V_5%_ < 5430 cGy
**GTV**	V_99%_ > 7800 cGy
**PTV**	V_0%_ < 8190 cGy
	V_2%_ < 8100 cGy
	V_97%_ > 7650 cGy
	V_99%_ > 7410 cGy

All prostate VMAT plans were optimized initially with the AAA algorithm and recalculated with the AXB algorithm. In our TPS, the grid size can be up to 3 mm for AXB, and up to 5 mm for AAA. In order to investigate the trend of dosimetric and radiobiological parameters as the grid increases, a grid size of 1, 3, and 5 mm for AAA and 1, 2, and 3 mm for AXB were adopted.

### Dosimetric and radiobiological parameters

In order to evaluate the dosimetric and radiobiological parameters, cumulative dose-volume histograms (DVHs) were calculated for each plan. Dosimetric parameters such as median, mean, maximum, minimum dose and V_95%_ (percent volume irradiated by 95% of the prescription dose) for PTV were analyzed. V_95%_ of PTV was used as a measure of the target coverage in this study. To evaluate the target dose of each VMAT plan, homogeneity index (HI), conformity index (CI), and conformation number (CN) were calculated for PTV. HI was calculated by Eq (1):

$$HI=\frac{D2-D98}{D50}$$ (1)

Where D2, D98_,_ and D50 represent the dose to 2%, 98%, and 50% volume for the PTV, respectively. A lower HI means that the plan has a more homogeneous target dose. CI was calculated by Eq (2):

$$CI=\frac{V_{RI}}{TV}$$ (2)

Where V_RI_ is the volume of reference isodose on body, and TV is the physical volume of PTV. The CI refers to the degree of isodose conformity, and it is ideal for the CI to remain close to 1. To assess conformity to target dose and the healthy tissue irradiation, CN was evaluated by Eq (3):

$$CN=\frac{{TV}_{RI}}{TV}\times \frac{{TV}_{RI}}{V_{RI}}$$ (3)

Where TV_RI_ represents the PTV volume covered with the reference isodose. The first term of CN refers to the target coverage, and the second terms indicate the degree of delivered dose on normal tissue.

For OARs, dosimetric parameter included the median, maximum, and minimum dose and a set of V_x%_, which is the volume of the organ receiving x% or more of the prescription dose.

In order to investigate the radiobiological impact on the PTV and various OARs, the values of TCP and NTCP were calculated from the DVH of planning data using a different dose calculation grid size and algorithm [25]. Equivalent uniform dose (EUD) is defined as the dose that when distributed uniformly over a structure would produce the same effect as the dose specified by the DVH. EUDs were calculated using Niemierko’s phenomenological model [26] by Eq (4):

$$EUD={\left(\sum_{i=1}\left(v_{i}D_{i}^{a}\right)\right)}^{\frac{1}{a}}$$ (4)

The EUD model can be used in both PTV and normal tissue by applying different input parameters. The *a* is a unitless parameter derived specifically from normal tissue or tumor properties. The *v*_*i*_ represents the relative sub-volume of the i-th that received a dose of *D*_*i*_, in Gy units. Therefore, the sum of all *v*_*i*_ is equal to 1 in the above EUD formula. Differential DVHs were obtained from a given VMAT plan to obtain the *D*_*i*_ and *v*_*i*_ at each structure. NTCP and TCP are expressed by Eqs (5) and (6):

$$NTCP=\frac{1}{1+{\left(\frac{{TD}_{50}}{EUD}\right)}^{4\gamma_{50}}}$$ (5)

$$TCP=\frac{1}{1+{\left(\frac{{TCD}_{50}}{EUD}\right)}^{4\gamma_{50}}}$$ (6)

The TD_50_ is the tolerance dose for 50% complication probability within a specific time interval. The TCD_50_ is the tumor dose to control 50% of the tumor when irradiated homogeneously, and *γ*_50_ is a unitless parameter derived from the slope of the dose-response curve that is specific to the organ or tumor. Table 2 lists the input parameters for calculating TCP and NTCP, and these parameters were referenced to other studies [23, 25].

**Table 2:** Parameters used to calculate tumor control probability (TCP) and normal tissue complication probability (NTCP).

Type	Organ	*a*	γ50	TCD50 | TD50	α/β
**Tumor**	Prostate	-13	2.2	67.5	1.5
**Critical Organ**	Rectum	8.33	2.66	80	5.4
	Bladder	2	3.63	80	7.5
	Lt femur head	13	2.7	65	3
	Rt femur head	13	2.7	65	3

In order to evaluate the feasibility of grid sizes and algorithms in clinical practice, we calculated the deviations of the mean, median, maximum, and minimum dose to the PTV and OARs. The deviations were compared between the VMAT plans and evaluated with the dose calculation times. The deviations are expressed by Eq (7):

$$Deviation=\frac{\left|Ref-Eval\right|}{Ref}\times 100$$ (7)

Where, *Ref* represents the reference dosimetric parameter and *Eval* refers to the dosimetric parameters of evaluated plans. The parameters with the AXB and a 1 mm grid size (AXB1) were selected as *Ref*, which is the combination for the most accurate algorithm and dose grid in this study. AXB have been reported as superior dose calculation algorithm in heterogeneous region and the general consensus is that finer dose calculation grid would produce more accurate dose. *Eval* were rest of plans such as AAA1, AAA3, AXB2, et cetera.

To clearly show the dose difference according to the algorithms, the AXB1 and AAA1 plans of one patient were imported into the Computational Environment for Radiotherapy Research (CERR version 4.6) [27], and we subtracted the dose distribution of AAA1 from that of AXB1. CERR is a programming package of MATLAB (MathWorks, Inc., Natick, USA), and has many functions for radiotherapy research such as the CT slice viewer, contouring tool, DVH calculation, dose distribution subtraction tool, et cetera. In addition, we subtracted the distribution of the 3 mm grid from that of the 1 mm grid for both algorithms to evaluate the grid effect on the VMAT plan.

### Statistical analysis and comparison of dose calculation time

Statistical analysis were performed using SPSS Statistics 19 (IBM SPSS, Chicago, IL) to assess statistical significance between algorithm type and grid size. The Wilcoxon signed rank test [28] was used in this study, and differences were considered statistically significant at p-values < 0.05. The calculation time of all AAA and AXB plans were also recorded to investigate the feasible grid sizes and algorithms in prostate VMAT.

## Results

### Dosimetric comparison

**Fig 1:**
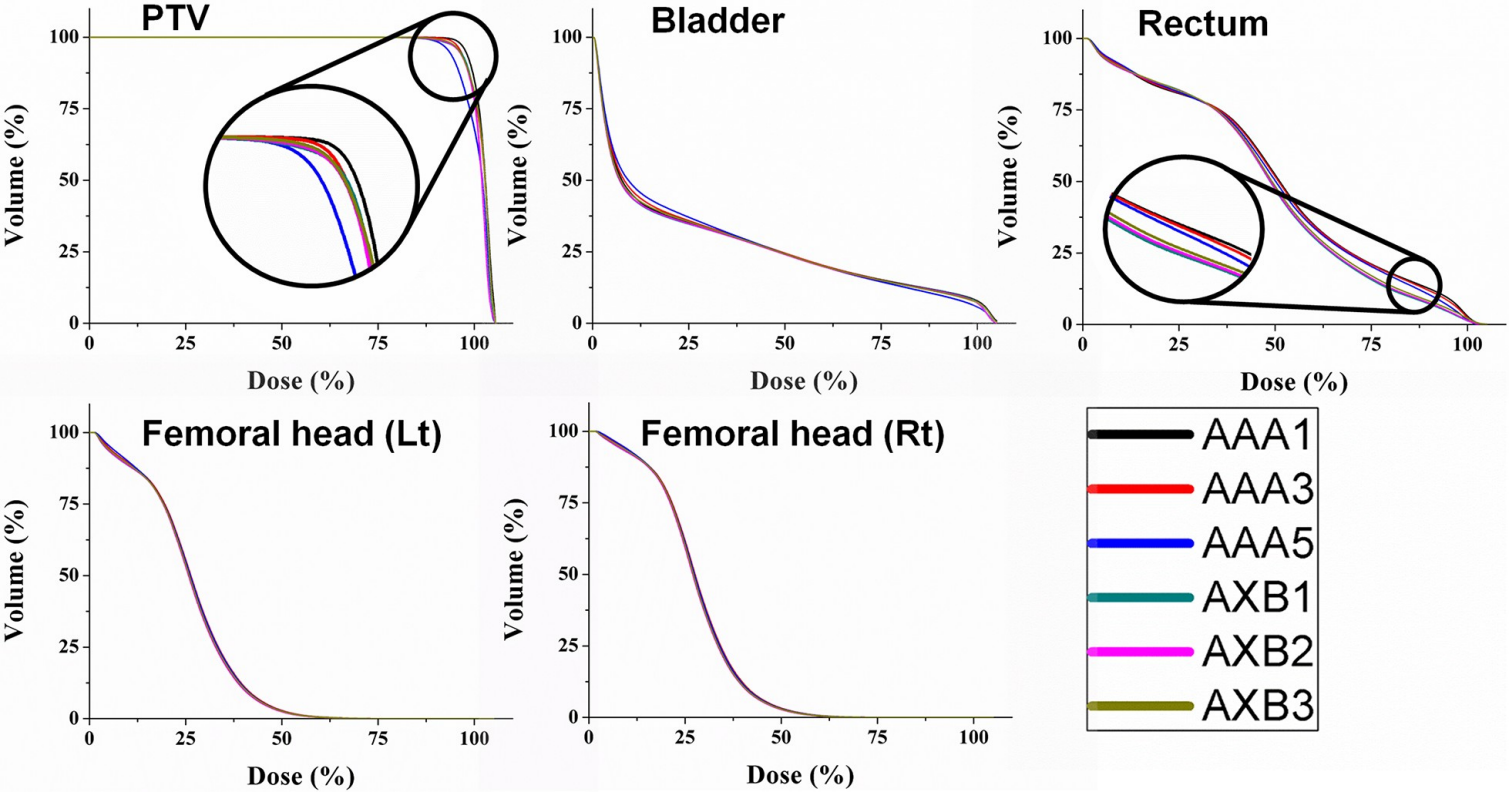
The average dose volume histograms of the planning target volume, bladder, rectum, and femoral heads on prostate VMAT plan using various calculation grid sizes and algorithms.

Table 3 shows the mean and standard deviation of dosimetric parameters to PTV on plans using different dose calculation grid sizes and algorithms. For PTV, the V_95%_ with the AXB1 was 96.65%, whereas the V_95%_ was 99.03% with AAA1. The difference in average V_95%_ between AAA1 and AAA3 mm was 1.22%, and that between AXB1 and AXB3 was 0.29%. The V_95%_ for AXB2 was 96.60%, and the V_95%_ difference between AXB1 and AXB2 was small (only 0.05%). The median dose slightly decreased over the change from AAA1 to AAA5. Contrary to the AAA trends, the median dose increased by 0.66 Gy (0.84% of prescription) over the variation from AXB1 to AXB3. A similar trend was observed for the mean and maximum dose of PTV over the transition from AAA1 to AAA5, and the mean dose decreased by 1.25 Gy accordingly. The CI value of AXB3 was 1.03, the closest to one in all cases. The HI of AAA5 was 0.14, which was the highest among all cases. Fig 1 shows the average DVH of PTV and different OARs on prostate VMAT plans using various calculation grid sizes and algorithms.

**Table 3:** Mean and standard deviation of dosimetric parameters for planning target volume (PTV).

	AAA−Mean(SD)			AXB−Mean(SD)		
	1 mm	3 mm	5 mm	1 mm	2 mm	3 mm
**V_95%_ (%)**	99.03 (0.43)	97.81 (0.42)	91.28 (1.15)	96.65 (0.73)	96.60 (0.72)	96.94 (0.71)
**D_median_ (Gy)**	80.25 (0.20)	80.16 (0.20)	79.66 (0.27)	79.54 (0.19)	79.53 (0.18)	80.20 (0.24)
**D_mean_ (Gy)**	79.85 (0.18)	79.51 (0.18)	78.60 (0.23)	79.10 (0.20)	79.01 (0.18)	79.53 (0.22)
**D_max_ (Gy)**	84.42 (0.70)	83.30 (0.39)	82.70 (0.53)	84.98 (0.76)	84.24 (0.73)	84.45 (0.77)
**D_min_** **(Gy)**	64.99 (5.13)	64.65 (4.77)	61.17 (3.87)	58.34 (4.95)	59.48 (4.69)	61.75 (4.84)
**CI**	1.09 (0.02)	1.04 (0.02)	0.93 (0.02)	1.04 (0.02)	1.03 (0.02)	1.03 (0.02)
**CN**	0.90 (0.01)	0.92 (0.01)	0.90 (0.01)	0.90 (0.01)	0.91 (0.01)	0.92 (0.01)
**HI**	0.09 (0.01)	0.10 (0.00)	0.14 (0.01)	0.12 (0.01)	0.11 (0.01)	0.11 (0.01)

SD: standard deviation

**Fig 2:**
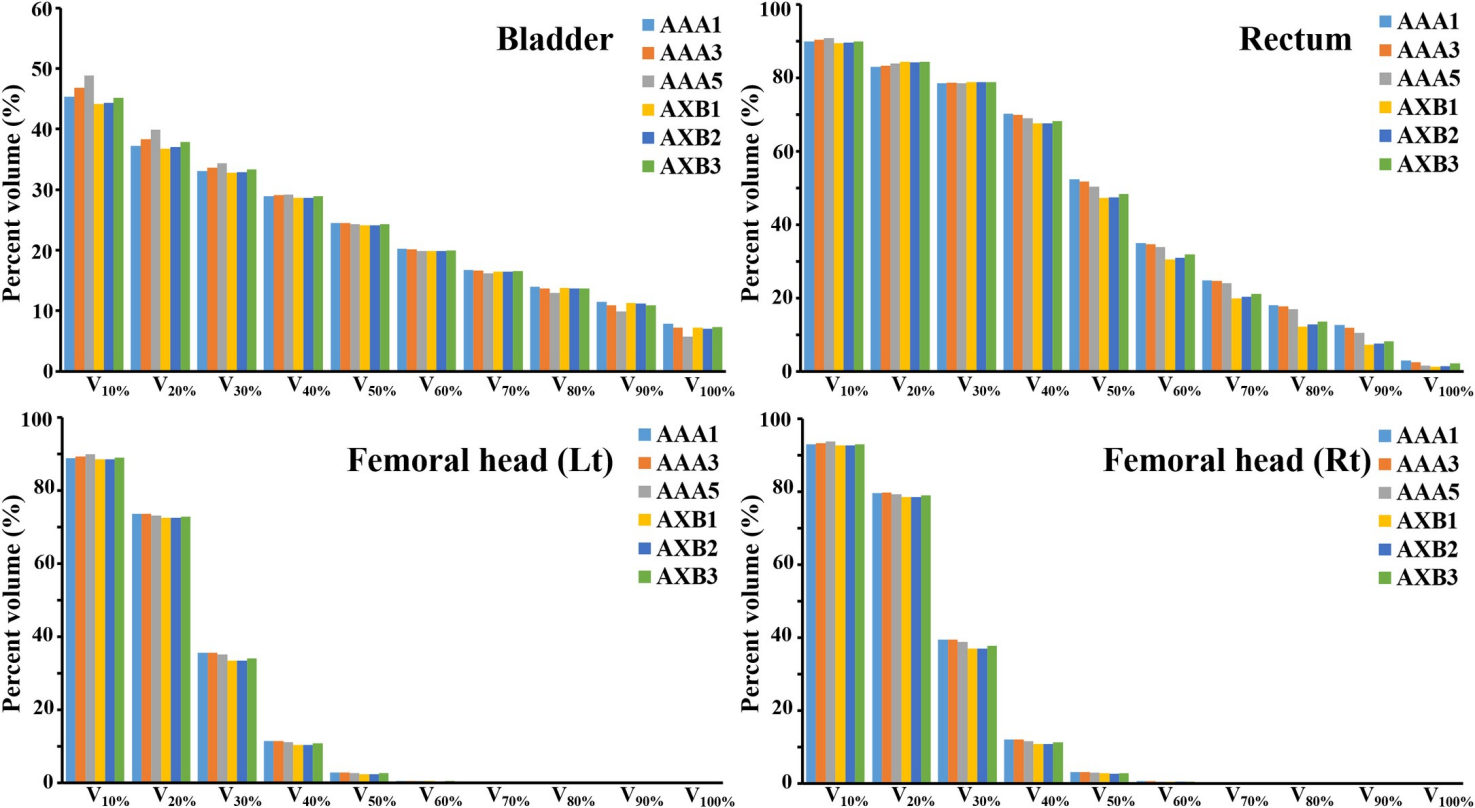
Percentage volumes receiving at least X% of prescription dose (V_X%_) of organs at risk.

For different OARs, the average dosimetric parameters are listed in Table 4, and the V_X%_ are shown in Fig 2. As shown in Table 4, the median and mean dose of rectum was higher in AAA1 than in AXB1, and the differences were 2.07% and 2.87% of prescription dose by simply subtracting those values in Table 4. Using a 1 mm dose grid, the difference of Vx% of rectum dose between the two algorithms was high in the region ranging from V_40%_ to V_90%_ (2.63% − 5.78%). V_20%_, V_30%_, and maximum dose was lower in AAA compared to AXB, and all other parameters were higher in AAA than in AXB at the same grid size. The difference in bladder dosimetric parameters for the two algorithms was less than 1% of the reference values, which were the bladder volume for Vx_%_ and the prescription dose for the other parameters. In left and right femoral heads, only V_20%_ to V_40%_ showed a difference greater than 1% of reference values between AAA1 and AXB1, and only V_30%_ showed a difference greater than 1% between AAA3 and AXB3. AAA predicted higher dosimetric parameters than AXB for both femoral heads.

**Table 4:** Dosimetric data of organs at risk.

		Bladder				Rectum			
		Maximum dose (%)	Minimum dose (%)	Mean dose (%)	Median dose (%)	Maximum dose (%)	Minimum dose (%)	Mean dose (%)	Median dose (%)
**AAA**	1 mm	107.33	1.40	27.97	14.27	104.54	2.33	51.69	49.14
	3 mm	106.19	1.44	28.18	14.83	103.71	2.38	51.50	48.94
	5 mm	105.18	1.47	28.27	15.53	103.01	2.42	50.91	48.37
**AXB**	1 mm	107.19	1.34	27.49	13.73	105.16	2.21	48.82	47.07
	2 mm	106.55	1.34	27.48	13.80	104.41	2.21	49.02	47.14
	3 mm	106.70	1.36	27.79	14.18	104.81	2.26	49.59	47.52
		**Right femoral head**				**Left femoral head**			
		Maximum dose (%)	Minimum dose (%)	Mean dose (%)	Median dose (%)	Maximum dose (%)	Minimum dose (%)	Mean dose (%)	Median dose (%)
**AAA**	1 mm	47.71	5.63	27.83	28.58	47.17	4.90	26.30	27.25
	3 mm	47.54	5.93	27.87	28.56	46.88	5.09	26.36	27.26
	5 mm	47.21	6.33	27.76	28.39	46.59	5.33	26.28	27.06
**AXB**	1 mm	47.11	5.27	27.25	28.00	46.48	4.64	25.74	26.71
	2 mm	47.11	5.36	27.25	27.99	46.44	4.67	25.74	26.71
	3 mm	47.23	5.58	27.46	28.19	46.42	4.81	25.95	26.89

The deviation of average dosimetric parameters depending on the algorithms and grid size is shown in Table 5. Almost AXB2 parameters were within 1% deviation, except the minimum dose to the PTV (1.95%) and right femoral head (1.71%). In the comparison with AXB3, the minimum dose of all structures except the bladder and the median dose to the bladder were different with AXB1 over 2%. However, most of the parameters of OARs were in excess of 2% different in comparison with AAA.

**Table 5:** The deviation of average dosimetric parameters depending on the algorithms and grid size. The reference is AXB plan with 1 mm grid, and the deviation is calculated by dividing the difference of dosimetric parameters between the reference and evaluation one by that of the reference.

			Maximum dose (%)	Minimum dose (%)	Mean dose (%)	Median dose (%)
**PTV**	AAA	1 mm	0.67	11.38	0.95	0.89
		3 mm	1.99	10.8	0.52	0.78
		5 mm	2.68	4.84	0.64	0.15
	AXB	2 mm	0.88	1.95	0.11	0.01
		3 mm	0.63	5.84	0.55	0.82
**Bladder**	AAA	1 mm	0.13	4.48	1.75	3.93
		3 mm	0.93	7.46	2.51	8.01
		5 mm	1.88	9.70	2.84	13.11
	AXB	2 mm	0.60	0.00	0.04	0.51
		3 mm	0.46	1.49	1.09	3.28
**Rectum**	AAA	1 mm	0.59	5.43	5.88	4.40
		3 mm	1.38	7.69	5.49	3.97
		5 mm	2.04	9.50	4.28	2.76
	AXB	2 mm	0.71	0.00	0.41	0.15
		3 mm	0.33	2.26	1.58	0.96
**Right** **femoral** **head**	AAA	1 mm	1.27	6.83	2.13	2.07
		3 mm	0.91	12.52	2.28	2.00
		5 mm	0.21	20.11	1.87	1.39
	AXB	2 mm	0.00	1.71	0.00	0.04
		3 mm	0.25	5.88	0.77	0.68
**Left** **femoral** **head**	AAA	1 mm	1.48	5.60	2.18	2.02
		3 mm	0.86	9.70	2.41	2.06
		5 mm	0.24	14.87	2.10	1.31
	AXB	2 mm	0.09	0.65	0.00	0.00
		3 mm	0.13	3.66	0.82	0.67

**Fig 3:**
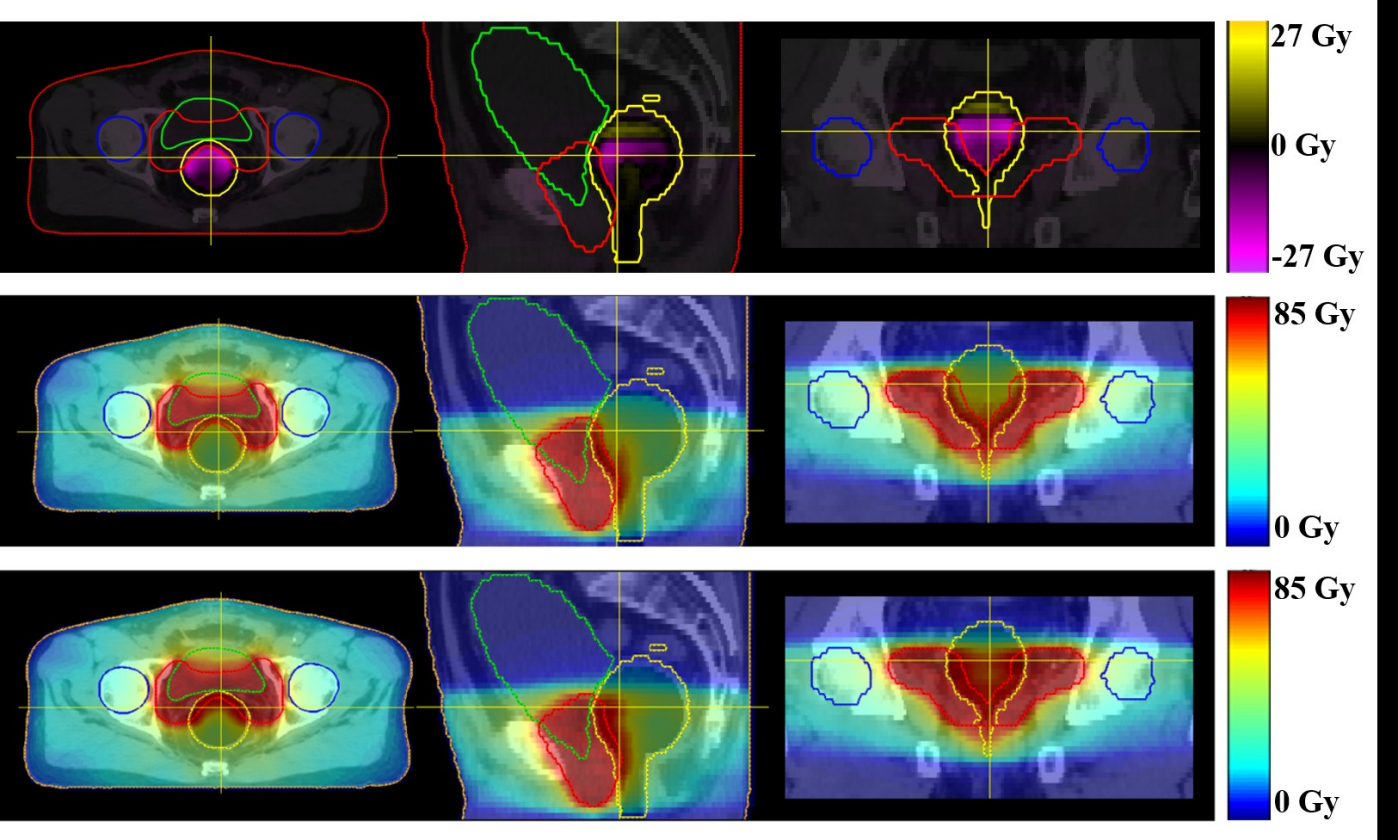
The dose distributions of one patient. The top line shows the dose distributions of the AXB plan minus AAA at 1 mm grid size. The median line shows the dose distributions of the AXB plan with 1 mm grid, and the bottom line shows the dose distributions of the AAA plan with 1 mm grid. The image planes of each line are axial, sagittal, and coronal view in that order.

**Fig 4:**
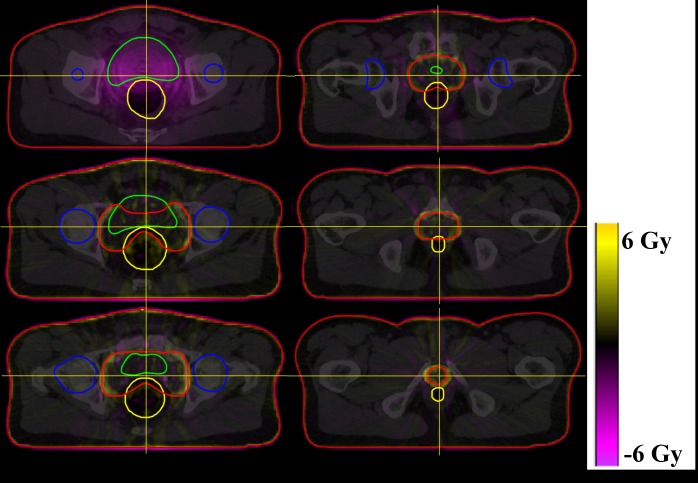
Dose distribution subtracted the plan with 1 mm grid from that of 3 mm in AAA. Each image are axial plane of one patient from the top of planning target volume to the bottom of that at 12 mm intervals.

**Fig 5:**
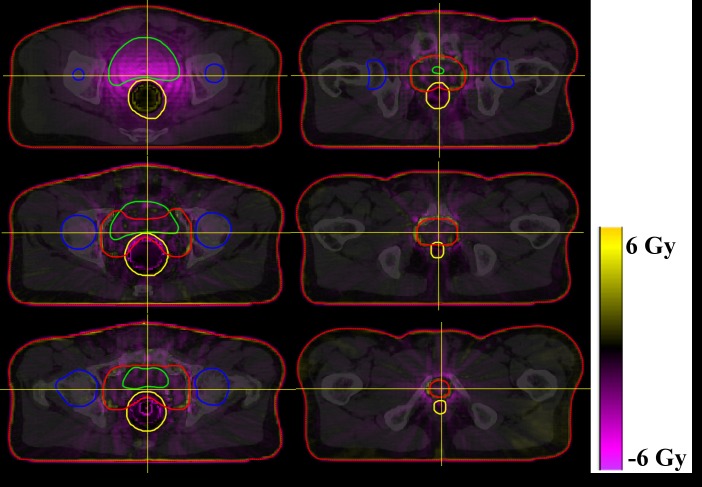
Dose distribution subtracted the plan with 1 mm grid from that of 3 mm in AXB. Each image are axial plane of one patient from the top of planning target volume to the bottom of that at 12 mm intervals.

The subtracted, AXB1 and AAA1 dose distribution of one patient are shown in Fig 3. Likewise, Figs 4 and 5 show the subtracted distributions between 1 and 3 mm dose grid plan of AAA and AXB, respectively. The red, blue, yellow, green, and orange lines refer to the PTV, femoral heads, rectum, bladder, and body contour, respectively.

### Radiobiological comparison

The average TCP and NTCP values with respect to algorithm and grid size are shown in Table 6. The difference of TCP values between AAA1 and AXB1 was 4.05%, and the difference between AAA3 and AXB3 was 0.9%. The largest NTCP of the rectum was 6.11% for AAA1, and the lowest NTCP was 3.23% for AXB1. In the bladder and the two femoral heads, no apparent NTCP values were observed, and all average NTCPs of the bladder and femoral heads were less than 1%, in all cases.

**Table 6:** Average and standard deviations of tumor control probability (TCP) and normal tissue complication probability (NTCP) values depending on the algorithms and grid size.

		AAA			AXB		
		1 mm Average (SD)	3 mm Average (SD)	5 mm Average (SD)	1 mm Average (SD)	2 mm Average (SD)	3 mm Average (SD)
**TCP (%)**	Prostate (PTV)	87.49 (2.02)	86.59 (2.14)	82.37 (3.07)	83.44 (3.06)	84.32 (2.44)	85.69 (2.23)
**NTCP (%)**	Bladder	0.05 (0.18)	0.05 (0.19)	0.04 (0.15)	0.05 (0.18)	0.05 (0.18)	0.05 (0.19)
	Rectum	6.11 (3.18)	5.55 (2.79)	4.77 (2.52)	3.23 (1.58)	3.42 (1.66)	3.99 (1.93)
	Rt femoral head	0.04 (0.11)	0.04 (0.11)	0.04 (0.10)	0.03 (0.08)	0.03 (0.09)	0.03 (0.09)
	Lt femoral haed	0.05 (0.14)	0.05 (0.13)	0.04 (0.12)	0.03 (0.10)	0.03 (0.10)	0.04 (0.11)

SD: standard deviation

### Statistical analysis and dose calculation time comparison

The p values for various comparative groups are shown in Table 7, and p values < .05 were observed in several dosimetric and radiobiological parameters. In the comparison between AXB1 and AAA1, most of the parameters had p-values of less than 0.05 and showed statistically significant differences. In the comparison between AAA3 and AXB3, the p-values of the mean and median PTV doses exceeded 0.05 compared to the 1 mm group. In the AAA plan comparison of grid size, a statistically significant difference between the 1 mm grid and the others were found for all PTV parameters. In OARs, the p-value of V_30%_ of rectum was higher than 0.05 according to grid size in the AAA case. The median dose and V_95%_ of the PTV for the AXB plans were not statistically significant between 1 and 2 mm grids, and a statistically significant difference was found for all rectum parameters between AXB1 and AXB2.

**Table 7:** Statistical analysis results depending on the dose calculation algorithms and grid size. The comparison groups are (AAA1 vs AXB1), (AAA3 vs AXB3), (AAA1 vs AAA3), (AAA1 vs AAA5), (AXB1 vs AXB2), (AXB1 vs AXB3) from the left.

	Algorithm	AAA vs AXB		AAA		AXB	
	Grid size	1 mm	3 mm	1 vs 3 mm	1 vs 5 mm	1 vs 2 mm	1 vs 3 mm
**PTV**	Mean dose	< .01	0.58	< .01	< .01	< .01	< .01
	Median dose	< .01	0.52	< .01	< .01	0.26	< .01
	TCP	< .01	< .01	< .01	< .01	< .01	< .01
	V_95%_	< .01	< .01	< .01	< .01	0.16	< .01
	HI	< .01	< .01	< .01	< .01	< .01	< .01
	CI	< .01	< .01	< .01	< .01	< .01	< .01
	CN	0.391	0.852	< .01	< .01	< .01	< .01
**Bladder**	Mean dose	< .01	< .01	< .01	< .01	0.56	< .01
	Median dose	< .01	< .01	< .01	< .01	0.01	< .01
	Maximum dose	0.42	0.01	< .01	< .01	< .01	< .01
	V_30%_	< .01	< .01	< .01	< .01	< .01	< .01
	V_50%_	< .01	< .01	0.25	0.01	< .01	< .01
	V_70%_	< .01	< .01	< .01	< .01	0.09	0.37
	NTCP	0.59	< .01	< .01	0.26	0.03	0.52
**Rectum**	Mean dose	< .01	< .01	< .01	< .01	< .01	< .01
	Median dose	< .01	< .01	< .01	< .01	0.02	< .01
	Maximum dose	0.01	< .01	< .01	< .01	< .01	0.01
	V_30%_	0.71	0.911	0.50	0.20	0.02	0.63
	V_50%_	< .01	< .01	< .01	< .01	< .01	< .01
	V_70%_	< .01	< .01	< .01	< .01	< .01	< .01
	NTCP	< .01	< .01	< .01	< .01	< .01	< .01
**Right femoral head**	Mean dose	< .01	< .01	< .01	0.02	1.00	< .01
	Median dose	< .01	< .01	0.16	< .01	0.32	< .01
	Maximum dose	< .01	< .01	0.02	0.01	0.90	0.51
	V_30%_	< .01	< .01	0.17	< .01	< .01	< .01
	V_50%_	0.04	0.04	0.35	0.43	0.35	0.08
	V_70%_	0.18	0.18	0.66	0.66	0.18	0.18
	NTCP	0.01	< .01	0.86	0.09	0.04	< .01
**Left femoral head**	Mean dose	< .01	< .01	< .01	0.40	1.00	< .01
	Median dose	< .01	< .01	0.59	< .01	1.00	< .01
	Maximum dose	< .01	< .01	< .01	< .01	0.38	0.72
	V_30%_	< .01	< .01	0.77	< .01	< .01	< .01
	V_50%_	0.04	0.04	0.89	0.04	0.08	0.04
	V_70%_	0.18	0.18	0.18	0.18	0.18	0.18
	NTCP	0.01	< .01	0.17	0.06	0.02	< .01

The average calculation times according to type of algorithm and grid size are shown in Table 8. With AXB, it took 4061 seconds to calculate the dose at the 1 mm grid size, which was 1850 seconds longer than the 2211 seconds of AAA. In the 3 mm grid, the calculation time difference between the two algorithms is 17 seconds.

**Table 8:** Average and standard deviation of dose calculation times recorded at different algorithms and grid size.

	AAA			AXB		
	1 mm Average (SD)	3 mm Average (SD)	5 mm Average (SD)	1 mm Average (SD)	2 mm Average (SD)	3 mm Average (SD)
**Time (s)**	2211 (155)	245 (27)	130 (10)	4061 (922)	671 (91)	262 (26)

SD: standard deviation

## Discussion

In several studies, AXB has been reported to predict more accurate doses in heterogeneous medium than AAA, considering tissue specific interactions of photons [29–31]. In our results, the V_95%_ of the PTV was higher in the AAA than the AXB, and the differences for both algorithms were statistically significant (p values < 0.01). By using the ERB, an air cavity is usually generated in the rectum structure, which may cause a region of overlap between the PTV and air cavity. As a result, the AAA predicted a higher dose to the air cavity within the PTV and could consequently estimate better target coverage than AXB. PTV may contain a portion of the ballooned rectum, where AAA overestimates the dose. However, this portion did not actually contain much dose, because this was air. Therefore, target coverage was estimated better than AXB when using the AAA.

Although the differences of median and mean doses to the PTV were statistically significant with respect to the type of algorithm at 1 mm grid size, these were less than 1% of the prescribed dose. At 3 mm grid size, the differences were not statistically significant and less than 1% of the prescription dose. The average V_95%_ of the PTV for the AAA plan decreased with increasing grid size, whereas V_95%_ for the plan with AXB increased slightly. For dose calculation, AXB discretizes the resolution in space, angle, and energy, and requires the material composition of voxels in the CT image by converting the Hounsfield unit to material derived mass density value. The automatic material composition is implemented with five biological materials divided according to the Hounsfield unit. When this material composition is performed on each dose voxel, the conversion of CT values might be affected by the size of the grid systematically. In our results, the p-value of the V_95%_ between AXB1 and AXB3 was less than 0.01, and the increased V_95%_ was 0.29% of the PTV volume. This means that the difference of dose grid size can produce systematic errors on target coverage of the PTV. However, it was not apparently changed. We think that the dose grid size may affect on each of the sub-procedure of the AXB dose calculation. Further study is needed to clarify these issues. The plans using AAA are more strongly influenced by the dose grid size compared to those using AXB. Due to the dramatic dose falloff near the PTV, the dose grid factors such as discrete sampling and volumetric averaging effects can work differently between both algorithms. As shown in Fig 4, a high dose difference was observed around the PTV, which is expressed in yellow. In general, AAA1 achieves a more precise dose prediction than AAA3 by using a fine dose grid, and this caused a sampling and volumetric averaging effect. Therefore, AAA1 may have a higher dose in the dose falloff region than AAA3. In comparison to Figs 4 and 5, the grid effect appears to have a relatively larger influence on AAA than on AXB around the PTV, which affects target coverage relative to the grid size.

To compare plan qualities in greater detail, the CI and HI showed statistically significant differences according to algorithms using the same grid size. The average CI of AAA was higher than that of AXB and far from the ideal value of one. This means that the volume above the prescribed dose is greater than the target volume when using AAA, and treatment plan could be evaluated to have worse conformity with AAA than AXB. However, the HI of AAA was less than that of AXB, and the AAA plan evaluations were more homogeneous than those of AXB. This, specifically, was the reason for the low dose in the air cavity of the PTV when using AXB. High doses in PTV air caused the increase in D98, leading to the decrease in HI. However, the dose overestimated by AAA in PTV air was not appropriate, and HI reduction of AAA did not guarantee the quality of a better treatment plan. Finally, AAA and AXB did not significantly differ with respect to the CN at the same grid size, as the average value was not apparently different between both algorithms.

As shown in Fig 3, the rectal dose was higher in AAA1 than in AXB1, and the dose of overlapped region between the PTV and the rectum was also higher in AAA1 compared with AXB1. In the subtracted dose distributions, dose difference between AAA1 and AXB1 were apparently large in only the rectum region, and AXB1 showed higher doses than AAA1 in the upper region of the rectum. Other studies have reported that the accuracy of AXB was superior to that of AAA in low density media [14, 15, 17, 18] and that AXB predicted higher doses of penumbra than the AAA in the air cavity [32, 33]. Based on the LBTE, AXB can consider the transport of photons and electrons through matter. Unlike AAA that uses a kernel of water with simplified density scaling for heterogeneous region, AXB use material library including five biologic materials (lung, adipose tissue, muscle, cartilage, and bone) and 16 non-biologic materials with a maximum density of steel. By using this, AXB can reflect characteristics of a various materials on dose calculation. Therefore, AXB is possible to correctly estimate the dose to a heterogeneous region such as the rectum. However, the dose calculation of AAA was associated with lateral and depth-directed components [34], and this algorithm cannot properly approximate the dose in the air due to the use of lateral scattering terms derived from Monte Carlo simulations in water [17]. The AAA compensates the heterogeneity by the inhomogeneity correction factor based on media density. As a result, AXB1 showed the predicted dose more correctly and estimated the higher rectum dose of the outside field, in comparison with AAA1.

The dosimetric difference of rectum was the largest among all OARs, and this largely depended on the algorithm. The average median and mean doses to the rectum were more than 2% higher in AAA1 compared with AXB1, and this dosimetric discrepancy has been reported to significantly influence radiobiological evaluation [35]. Likewise, we observed that the average NTCP in the rectum was heavily influenced by the respective algorithm. The rectum V_40%_ to V_90%_ showed large differences that depended on the algorithm rather than the dose grid. This was caused by the overlap between the PTV and rectum, which was induced in the optimization process to receive a high dose. In the bladder, most parameters showed statistically significant changes with both algorithms, however the changes were less than 1% of reference. Both femoral heads showed significant differences according to the algorithm and grid size, however the differences were not apparent.

In our results, the TCP differences according to algorithm and grid size were statistically different, and the TCP decreased as grid size increased with AAA. In contrast, the TCPs of AXB1 and AXB2 showed similar values, and the TCPs increased about 1%, in comparison with AXB1, as dose grid size increased to 3 mm. Under the same grid size, the average TCP of AAA was higher than that of AXB. By using the AAA, the target coverage (V_95%_) was superior to that of AXB. Therefore, the calculated TCP of AAA was relatively higher than that of AXB, and the TCP differences among the algorithms showed a quite large variations (4.05%), which were statistically significant (p-value <0.01). We used EUD-based TCP models. As described above, AAA predicted doses with overestimated errors in the air inside PTV than for AXB. This leads to the increase in EUD and TCP of AAA compared with AXB. The NTCP difference of the rectum was the largest among all the OARs, and this depended on the algorithm. The average NTCP of AAA was higher than that of AXB, and the average NTCP of the rectum decreased with increasing dose grid in the case of AAA, and inversely increased with AXB. In addition, all p-values of the rectum were less than 0.01, depending on the algorithm and grid size. This means that the rectum was influenced significantly by both algorithm and dose grid. Although we performed the statistical analysis depending on the algorithms and grid sizes, the impact of these factors on the bladder and both femoral heads was not clearly predictable due to very low NTCP values.

Rectal complications in radiation therapy are mainly caused by rectal tissue damage, and the dose deposited in air may not contribute to rectal toxicity from a clinical viewpoint. Our results for NTCP and dosimetric parameters in rectum contoured with air include may not have a direct impact on the clinical significance. Streller et al. [36] evaluated the impact of ERB on rectal wall dose by using AAA algorithm. However, dosimetric properties of two algorithms were different at the interface between air and matter, and the AXB showed more similar dose distribution to film measurement than AAA at the interface [33]. In addition, the dose rebuild-up has been reported with AXB [33]. The dose rebuild-up causes a large dose increase from air to matter, which can have a significant impact on dose calculations. Therefore, as a future study, additional analysis of the rectal well according to the algorithm and grid is necessary to perform monitoring of the control/complications of patients.

The International Commission on Radiation Units recommends 5% of overall absorbed dose delivery accuracy, and the American Association of Physicists in Medicine recommends a 2% dose calculation accuracy [35, 37]. Considering the calculation time, the use of a 1 mm grid is not feasible in the clinical practice. In the previous reported studies, the use of a 2.5 mm grid should be recommended to reduce the dose distribution error, and 2 mm should be recommended at least in high dose gradients [38, 39]. Therefore, from our results, we recommended a 2 mm grid with AXB, considering the relatively short dose calculation time and calculation accuracy for prostate VMAT.

## Conclusion

The AXB and AAA showed statically significant difference in dosimetric and radiobiological parameters. Our study demonstrated that the calculated grid size worked sufficiently enough to influence the plan quality evaluation. As previously reported, the AXB and fine dose grid provided significantly accurate dose calculations in air cavity regions, and our results indicated that AXB2 had sufficient accuracy and was reasonable, in terms of dose calculation time, compared the AXB1. So, we suggest employing AXB algorithm and 2 mm grid for improving treatment efficiency of VMAT plans for prostate cancer.
